# Prehospital nursing students' experiences of patient safety culture in emergency medical services—A qualitative study

**DOI:** 10.1111/jocn.16396

**Published:** 2022-06-07

**Authors:** Anu Venesoja, Veronica Lindström, Maaret Castrén, Susanna Tella

**Affiliations:** ^1^ South Carelia Social and Healthcare District Lappeenranta Finland; ^2^ Department of Emergency Care and Services University of Helsinki and Helsinki University Hospital Helsinki Finland; ^3^ Division of Nursing, Department of Neurobiology, Care Sciences, and Society Karolinska Institutet Stockholm Sweden; ^4^ Samariten Ambulance Stockholm Sweden; ^5^ Department of Health Promotion Science Sophiahemmet University Stockholm Sweden; ^6^ LAB University of Applied Sciences Lappeenranta Finland; ^7^ University of Eastern Finland Kuopio Finland

**Keywords:** ambulance service, emergency medical services, patient safety, prehospital nursing student, safety culture, safety management

## Abstract

**Aims and Objectives:**

To describe prehospital nursing students' experiences of patient safety culture in emergency medical services during their internship.

**Background:**

Patient safety culture in the emergency medical services is a complex phenomenon including more than organisational policies and practices and professionals' technical skills.

**Design:**

The descriptive qualitative approach used the Sharing Learning from Practice to improve Patient Safety Learning Event Recording Tool, which includes both open‐ended and structured questions.

**Methods:**

Purposeful sampling was used, and data were collected from graduating prehospital nursing students (*n* = 17) from three Finnish Universities of Applied Sciences. Open‐ended questions were reviewed using thematic analysis, and frequencies and percentages were derived from structured questions. COREQ guidelines were used to guide this study.

**Results:**

Four themes were identified during the thematic analysis: environmental and other unexpected factors in emergency medical services, working practices and professionalism in emergency medical services, teamwork in emergency medical services and feelings related to patient safety events in emergency medical services. Patient safety events described by students were seldom reported in the healthcare system or patient files. According to the students, such events were most likely related to communication, checking/verification and/or teamwork.

**Conclusions:**

This study shows that prehospital nursing students can produce important information about patient safety events and the reasons that contributed to those events. Therefore, emergency medical services organisations and managers should use students' observations to develop a patient safety culture in emergency medical services.

**Relevance to clinical practice:**

Understanding how prehospital nursing students have experienced patient safety culture during their internships on ambulances can support educational institutions, together with emergency medical services organisations and managers, to improve policies for students to express patient safety concerns as well as patient safety successes.

1

**What does this paper contribute to the wider global clinical community?**

- Prehospital nursing students can share detailed information about patient safety events and reasons that contribute to those events.
- Students are future employees, so EMS organisations and managers should use students' observations to develop patient safety culture.
- Students need support to discuss and reflect on patient safety events they have observed; otherwise, patient safety events could affect students' future careers and professionalism.
- More work is required on how educational institutions, EMS organisations and managers should work together to enhance opportunities for students to express their concerns about patient safety events as well as to celebrate successes.

## 2 INTRODUCTION

This article describes prehospital nursing students' experiences of patient safety culture in emergency medical services (EMS) during their internships in ambulance service. In many ways, the EMS working environment is different than other healthcare settings. For example, EMS personnel must work outside in different conditions, in patients' homes and inside moving ambulances. In general, EMS working conditions are not as stable as in other healthcare settings. Therefore, prehospital nurses need different kinds of knowledge compared with nurses working in hospitals (Holmberg et al., 2017). Another difference compared with the hospital working environment is that EMS personnel usually work in pairs, and usually doctors are not physically available on‐site. These special features, typical of EMS work, could cause harm to patients but also to prehospital nurses or other EMS personnel (Bigham et al., 2012).

The competences and educational demands needed to work in the EMS differ around the world. Some countries use emergency medical technicians and paramedics; other countries, such as Sweden, Belgium and Finland, staff the ambulances with registered nurses (RNs) (Sjölin et al., 2019). Finnish prehospital nursing education has similarities with other countries' education systems, but it is not totally comparable, for example, with education in the other Nordic countries or the UK (Dúason, Ericsson, et al., 2021; Paramedics, 2019). In Finland, prehospital nursing studies mainly focus on prehospital nursing, but they also include RN qualification. The extent of the Finnish prehospital nursing studies is the 240 European Credit Transfer and Accumulation System (ETCS; 4 years/6480 h; 1 ETCS = 27 h). This 240 ETCS includes 80–90 ETCS internships, whereof approximately 30 ETCS include internships in ambulance service.(LAB, 2022; Metropolia, 2022) After graduation Finnish prehospital nurses can work in advanced level ambulances or other health care settings as RNs.

## 3 BACKGROUND

One definition of patient safety culture is ‘the willingness and ability of an organisation to understand safety (and the hazards) as well as the willingness and ability to act on safety’ (Reiman et al., 2010). It is also important to mention organisation culture in healthcare because organisation culture in healthcare has been shown to be connected to quality of health care but also to patient safety. After all, organisation culture in health care includes the same three levels: visible manifestations (organisational dimension in patient safety culture theory), shared ways of thinking, including beliefs and values (social processes in patient safety culture theory) and deeper shared assumptions (psychological dimension in patient safety culture theory) (Mannion & Davies, 2018; Reiman et al., 2010). When these three levels of culture are put into the EMS context, visible manifestations include, for example, working conditions, communication styles and the EMS guidelines (organisational dimension). In turn, shared ways of thinking mean, for example, beliefs and values typical for EMS personnel (social processes). Deeper shared assumptions determine, for example, perceptions, thought processes, feelings and behaviour manifesting in EMS assignments (psychological dimension) (Forsell et al., 2020).

Some may argue that patient safety research in EMS mainly focuses on visible manifestations of EMS patient safety culture. For example, a previous study asserts that key issues of patient safety are technical skill, competence and factors related to EMS working environments (Atack & Maher, 2009). Patient safety issues in EMS are mainly focused on clinical judgement, adverse events and error reporting, communications, ground vehicle safety, aircraft safety, interfacility transport, field intubation (Bigham et al., 2012) and non‐conveyed patients (Lederman et al., 2021; Paulin et al., 2021). Teamwork has also been recognised as a visible manifestation of a patient safety culture (Reiman et al., 2010; Sammer et al., 2010). For example, in the EMS context, it has been shown that the most common reason for adverse events is actions or inactions by the team (Hagiwara et al., 2019). So, while teamwork is also a visible manifestation of culture, it also raises a question of what other factors are behind these visible manifestations?

Psychological safety, which basically means that everyone can speak up without fear of belittling or other negative consequences, has been recognised as a part of patient safety culture and teamwork (Edmonson, 2004; Sammer et al., 2010). For example, it has been shown that the prevailing patient safety culture has a positive impact on organisational support (Zhang et al., 2019). The same study showed that patient safety culture and organisational support have a positive effect in reducing the second victim‐related distresses. The term ‘second victim’ has been used to describe healthcare personnel who have been affected psychologically or physically after they have (potentially) caused harm to their patients (Wu et al., 2020).

One sign of a lack of teamwork and psychological safety could be if despite knowledge of patient safety issues in the EMS, there are still barriers to reporting patient safety events in EMS caused mainly by a fear of consequences—for example, fears of being punished, suspended, terminated, investigated by national authorities or decertified (Sinclair et al., 2018). From the patient safety point of view, it has also been found that managers' own behaviour can influence the quality of care and adverse events (Labrague, 2021). On the contrary, the way patients are treated during the EMS mission and how they are kept informed affect their experience of safety (Péculo‐Carrasco et al., 2020; Venesoja et al., 2020). These could be seen as a manifestation of levels of shared ways of thinking (or social processes) but also a collective sensemaking and deeper shared assumptions (or psychological dimension) and the effect of both levels on patient safety.

All in all, patient safety culture is a complex phenomenon, and it is good to notice that culture change interventions are rarely successful if the changes are forced from the top down. From the EMS managerial point of view, the easiest way to affect the culture is to take care of working conditions, including vehicles, equipment, stations and career development (Wankhade & Brinkman, 2014). On the contrary, learning is a part of a patient safety culture (Reiman et al., 2010; Sammer et al., 2010), and part of people's learning is ‘constructing knowledge and meaning from real‐life experience’ (Yardley et al., 2012). For example, it has been shown that in the workplace, novices—for example, students or new graduates—learn from experienced workers and vice versa (Brooks et al., 2020). Even though the aim of prehospital nursing students' clinical training is to apply theory to practice, clinical training also offers students experiences of patient safety culture in EMS.

It is important to offer managers and EMS personnel knowledge about how prevailing patient safety culture among EMS personnel appears from prehospital nursing students' point of view. This adds to managers' and EMS personnel's knowledge about both good and bad manifestations of patient safety culture in EMS. Because patient safety culture affects the safety of the patients (Reiman et al., 2010), knowledge concerning that culture in EMS can help to enhance patient safety at the organisational level, team level and even individual level. Therefore, the aim of this study was to describe prehospital nursing students' experiences of patient safety culture in emergency medical services during their internships.

## 4 METHODS

### 4.1 Study design

The study used a descriptive qualitative approach, and data were collected through written descriptions by using the Sharing Learning from Practice to improve Patient Safety Learning Event Recording Tool (Steven et al., 2021). Consolidated criteria for reporting qualitative research were used to guide this study (Tong et al., 2007); see Appendix S1.

### 4.2 Setting

In Finland, eight Universities of Applied Sciences (UASs) educate prehospital nurses (bachelor's level). Finnish prehospital nursing studies include patient safety education. It can be a part of all the prehospital nursing studies, or it can be organised as a specific patient safety course. Before internships in the ambulance service, Finnish prehospital nursing students complete internships in other healthcare settings, such as medical and surgical wards (LAB, 2022; Metropolia, 2022).

All the UASs are located in urban areas. However, prehospital nursing students do their internship periods in different healthcare districts. Depending on an EMS organisation's area of operation, it could cover urban, rural or both areas. According to the official statistics, depending on the size of a healthcare district area, the number of annual EMS tasks can vary between a little under 8000 and over 316,000 tasks per year. The emergency response centre's official statistics show that most of the EMS calls are non‐urgent and are accounted to medical reasons or falls.

Prehospital nursing students' first internship in the ambulance service is basic‐level training (basic life support techniques), and the final internship in the ambulance service contains more on advanced life support techniques and strengthens the students' decision‐making capacity. It is possible that during some internship periods in the ambulance service, prehospital nursing students can be supervised by professionals other than prehospital nurses. Currently, EMS in Finland can also work, for example, as emergency medical technicians, firefighters or nurses with or without special training in prehospital care. Therefore, we have used the term ‘EMS personnel’ to cover all these different professional titles.

### 4.3 Instrument

The Sharing Learning from Practice to improve Patient Safety Learning Event Recording Tool was used for data collection. This learning event recording tool uses the critical incident technique (CIT) methodology where participants are guided to memorise and describe their observations concerning critical incidents (positive or negative) (Flanagan, 1954). CIT is widely used as a learning tool in nursing and healthcare professional education as well as in personal reflection (Steven et al., 2020). The Learning Event Recording Tool was developed by the Sharing Learning from Practice to improve Patient Safety research team. The tool's validity and reliability was demonstrated in previous studies when researchers collected patient safety learning experiences from nursing students in five different countries and in different healthcare settings, including EMS (Steven et al., 2021; Steven et al., 2019; Tella et al., 2016). This recording tool is publicly available in various language versions in Sharing Learning from Practice to improve Patient Safety web pages (https://www.slipps.eu/slipps‐learning‐event‐recording‐tool‐slert/)

The Sharing Learning from Practice to improve Patient Safety Learning Event Recording Tool includes three parts, A, B and C. A and B were covered by open questions, and C with structured questions. In part A, the respondents were asked to describe a positive or negative patient safety event as accurately as possible. Writing the description was guided with supplementary questions: what happened, who was involved and what they did, when and where it happened, what the outcome/result was and if it was discussed with the persons involved. In part B, the respondents were asked to describe their feelings afterwards, what they learned from the event, and what they thought others should learn from the event. Part C included structured background questions where the respondent selects the most appropriate option(s) from those given. The background questions were unchanged: age, gender, year in programme, what the event broadly related to (communication; checking/verification; teamwork; leadership, guidance and education; handover/information transfer; procedure and/or treatment; moving and handling; decision making; using technology or equipment; medications; confidentiality; violence; food and nutrition; infection prevention and control; invasive procedures and other), what type the event was (good practice, near miss, hazard and adverse event) and whether it was reported through the reporting system and documented to the patient medical record. However, in this study, we research only patient safety events during internships in the ambulance service. Therefore, we left out two background questions: profession and the type of clinical/work placement in which the event happened

### 4.4 Participants and data collection

Prehospital nursing students were recruited from three UASs in southern Finland, and the inclusion criteria were that they be final year prehospital nursing students who have completed all internships in the ambulance service or doing their last internship in the ambulance service. Therefore, purposeful sampling was used (Suri, 2011) aiming to get descriptions from prehospital nursing students of patient safety culture after they had done several internships in the ambulance. Data collection started in spring 2020 and continued in autumn 2020 because the number of responses was small after the first round, possibly caused by the COVID‐19 disruption and students changing to distance learning. Data collection was carried out by using the Webropol® survey tool where students could write about more than one experience, if they desired. Researchers had no direct contact list/email addresses of all potential participants (limited in the research permits). The first author organised data collection. Practical matters and sharing the invitation to the prehospital nursing students were arranged with the students' course leaders/teachers. They sent the survey link and the invitation to participate to the students. In addition, each UAS was asked about the opportunity to present this study directly to the potential participants. However, because of the COVID‐19 pandemic, the possibility of face‐to‐face meetings with the students was limited. We used the opportunities offered, such as one UAS offering the opportunity to talk with the students in two online meetings, and another UAS offered the opportunity for face‐to‐face data collection. We asked teachers to remind students to participate in our study once in the spring and twice in the autumn. After the second round of data collection, we read all the free‐text data and determined that we had enough information to replicate the study. Similar phenomena and themes recurred in the descriptions. Therefore, we evaluated that the saturation point was achieved and considered that as a criterion to discontinue data collection.

### 4.5 Analysis

The prehospital nursing students' written descriptions of patient safety events were analysed using thematic analysis (Braun & Clarke, 2006). Written data included 12 pages of free text (Calibri 12, spacing 1.5). Analysis was supported by using Atlas.ti® software. The first author and the last author read the text independently several times. The first author marked representative quotations in the text and made notes for the memo. The memo was checked and updated as necessary during the analysis process. After that the coding started. The codes were single words or short sentences. These codes were then collated into preliminary themes, and the last author presented her suggestions for the themes. Then, the first and last authors reviewed the coded themes together. The authors continued analysing the data together and recognising the themes until consensus was reached. To ensure that the themes were credible and described the phenomenon, the first author presented the preliminary results to other prehospital nursing students (*n* = 12) not from the participating UASs. These students highlighted the meaning of teamwork in the EMS field. With less discussion, the students approved the other preliminary themes. However, authors continued the analysis to achieve a more coherent result. Analysis continued by checking and evaluating the themes together with all authors. During analysis, there was back and forth movement between the produced data analysis, coded extracts and the entire data set. The endpoint of this analysis was the finalised report. An example of the coding tree is presented in Appendix S2.

From the structured data, the count of factors affecting to the patient safety events was calculated. Factors were counted from the students' responses to the question ‘what was the event broadly related to’ (options defined in Sharing Learning from Practice to improve Patient Safety Learning Event Recording Tool). Responses from the question ‘reporting afterwards’ were also counted.

### 4.6 Ethical considerations

In this study, the guidelines of the Declaration of Helsinki were followed (World Medical Association, 2013). We consulted the Helsinki University Hospital ethics committee before conducting the study, and they stated that Finnish law does not require a formal ethics review for this kind of study design. Before the study, we obtained research permits from all three UASs and the Sharing Learning from Practice to improve Patient Safety research team to use the tool. Accurate identifying information (e.g., name, date of birth, address, IP address or internship organisation information) was not collected for the participants. In connection with the open‐ended questions, the participants were reminded not to ‘use any names of people or healthcare organisations’. Participation was voluntary, and writing and sharing the experience were considered as consent to participate in the study. Participants were informed that after they had submitted their responses, those responses would be used as a part of this study. A data protection statement and information about the study's purpose and researchers (females) were shared to the participants together with the survey link. Therefore, the need for written informed consent was waived. The participants received no incentives. The participants had the right and possibility to withdraw from the study by suspending their writing or electing not to send the written experience. The participants were informed that participation/refusing to participate had no effect on their studies.

## 5 RESULTS

A total of 18 descriptions of patient safety events were described by the prehospital nursing students; one description was not included because it was not from an internship in the ambulance service. Therefore, 17 descriptions were included in the analysis. Eight of the participants were male and eight females and one participant chose not to answer. In total, 14 of the participants were 30 years or under; the rest of the participants were over 30 but under 40.

Most of the patient safety events were near misses (8) or hazards (4). Out of the descriptions, one student wrote that the patient safety event was reported via the healthcare reporting system, and none of the students saw that event reported to patient medical records. The patient safety events students encountered during their ambulance internships were complex, and based on students' views, appeared to contain more than one factor associated with one patient safety event. For example, one student (S9) chose five factors associated with patient safety events (communication; decision making; leadership, guidance and education; moving and handling and teamwork) in one patient safety event. All the factors associated with patient safety events, based on students' views, are presented in Table 1.

**TABLE 1:** Patient safety events and what the event was related to, chosen by students

Experienced event type^a^	*N* = 17 (100%)
Good practice	*n* = 5 (29.4%)
Near miss	*n* = 8 (47.1%)
Hazard	*n* = 4 (23.5%)
Adverse event	*n* = 0 (0%)
Reported through a healthcare reporting system^a^	
Yes	*n* = 1 (6%)
No	*n* = 6 (35.2%)
I do not know	*n* = 8 (47.1%)
N/A	*n* = 2 (11,7%)
Incident documented in the patient files^a^	
Yes	*n* = 0 (0%)
No	*n* = 7 (41.2%)
I do not know	*n* = 8 (47.1%)
N/A	*n* = 2 (11.7%)
Where event was related to^a^	
Communication	*n* = 11 (64.7%)
Checking/verification	*n* = 8 (47.1%)
Teamwork	*n* = 6 (35.2%)
Leadership, guidance, and education	*n* = 5 (29.4%)
Handover/information transfer	*n* = 5 (29.4%)
Procedure and/or treatment	*n* = 5 (29.4%)
Moving and handling	*n* = 5 (29.4%)
Other	*n* = 4 (23.5%)
Decision making	*n* = 3 (17.6%)
Using technology or equipment	*n* = 3 (17.6%)
Medications	*n* = 3 (17.6%)
Confidentiality	*n* = 2 (11.7%)
Violence	*n* = 2 (11.7%)
Food and nutrition	*n* = 0 (0%)
Infection prevention and control	*n* = 0 (0%)
Invasive procedures	*n* = 0 (0%)

^a^ Students assigned.

**FIGURE 1:**
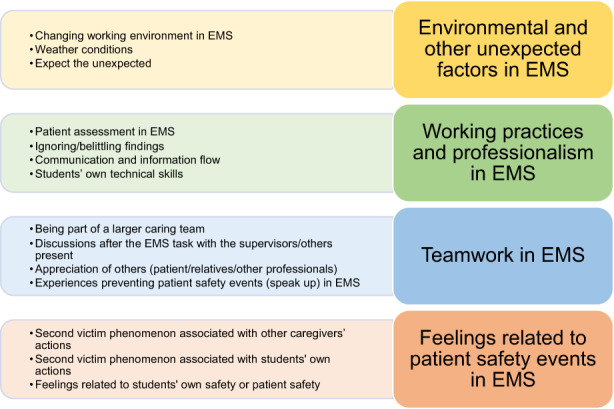
Prehospital nursing students’ experiences related to patient safety culture in EMS

Four themes were identified during the thematic analysis of the free text data: *environmental and other unexpected factors in EMS, working practices and professionalism in EMS, teamwork in EMS* and *feelings related to patient safety events in EMS*. Collated extracts within themes are presented in Figure 1. Themes are discussed in more detail with illustrative quotations below.

### 5.1 Environmental and other unexpected factors in EMS

Students described how a complex working environment and the unexpected are part of work in the EMS. When students described their experiences of environmental and other unexpected factors, they related how changing working conditions affect patient safety and/or occupational safety. For example, according to the students' experiences, circumstances at the patient home or outside where the patient was waiting for the ambulance caused, in some cases, a threat to the patient but also occupational safety.

Ground was difficult, and it threatened stable patient transport. We have to stop several times because of stumps, sticks and potholes. (S9).

In some cases, students experienced situations in which patients' current or previous behaviour was a threat to the patients themselves and/or to the EMS personnel. This did not automatically lead them to fear for their own or the patients' safety, but mainly reminded them to expect the unexpected, described briefly as the ‘+1 rule’.

We turn the man to his back and then we saw a gun in his hand. Quickly we string together that the patient has [been] shot in his head. (S8).

### 5.2 Working practices and professionalism in EMS

In the patient safety events recounted by students, they reflected on their experiences conducting patient safety working practices and professionalism. Based on students' views of working practices and professionalism, behaviour and attitudes affected the situation, including how the EMS or other healthcare personnel used their professional skills and how that affected the patient assessment. This kind of reflection recalled situations in which the supervisors or the consulting doctor ignored the other professionals' observations or situations where the patient was assessed incompletely. Students started to reflect on other professionals' behaviour in situations in which the students experienced their supervisor or some other healthcare professional jeopardising patient safety in their actions.

They have not taken essential measurements, which are very easy to take. Potentially life‐threatening condition. Even though it is nice to go home in time, it is still important that you take all measurements as instructed. (S12).

Students also described how EMS professionals' actions affected their views of their own professionalism. In some situations, students experienced, in their point of view, their supervisors or other EMS professionals acting unprofessionally and neglecting proper patient assessment. During and after these situations, students started to believe more strongly that every patient should be assessed properly. For example, when students recognised other EMS professionals' unprofessional behaviour, the students resolved that they did not want to act like that or wondered why EMS professionals did not want to do their jobs well.

In my opinion, the course of the situation affected my supervisor's bad attitude towards substance abuse users (it has shown already in the previous EMS missions). I learned that I have to see behind the substance abuse and take seriously all the symptoms and other things, what patient and next of kin tell us, and then do the careful assessment. (S4).

Or as one of the students stated,

Experience gets me to think about my own professionalism and professional pride. Why can't you do [the job] properly right away, professionally, friendly and serve the patient. (S11).

Students experienced that when they or their observing colleagues do careful assessment, it has an impact on patient safety. They described how careful assessment has helped them recognise those patients needing urgent treatment or another kind of attention from the EMS personnel. Especially, students' experiences of observing colleagues' persistence trying to understand the patient's situation amazed the students. At the same time those experiences strengthened students' perceptions and understanding of safe working practices.

At the end, what I remember was that my supervisor did not give up easily when she/he knows that patient didn't survive alone at home. She/he want to know the reason why the patient did not want to go to the hospital because she/he wants to persuade this patient to the hospital (S10).

Students described that clear communication was an important part of patient safety working practices, but also in occupational safety working practices. Clear communication helps students experience good working practices. On the contrary, experiences where there were failings in asking for or receiving information, too, help students understand why clear communication and smooth information flow is important. Those experiences can help them remember the value of good communication in the future.

I drew attention to the importance of reporting and how to confirm that recipient has [understood] that. Sometimes it goes that even [if] you give comprehensive report, it is not listened or internalised. (S6).

### 5.3 Teamwork in EMS

From the students' descriptions, experiences of being part of the team, discussions afterwards and appreciation of others (other professionals and patients) were issues identified as experiences of conducting teamwork. Mainly the students had experienced being an equal part of a team. This appears when students describe their experiences of shared situational awareness during a task or when they have experienced being part of a team in other ways during the task.

In the beginning we treat the patient together with my other supervisor and two firefighters. (S8).

Experiences of teamwork and being part of the team were strengthened if the event was discussed afterwards with the supervisors or the whole team who were involved.

After the event, we first discuss it together with my supervisor, who told me that I was acting right when I followed the woman. It was nice to get positive feedback for my own performance. (S14).

Even though students had positive experiences of teamwork, the teamwork climate in the EMS was not so strong that students had courage to speak up in a situation by themselves. For example, sometimes, teamwork does not happen properly. In some situations, supervisors gave more responsibility to the student but also a lack of support. This kind of behaviour from the supervisors led to the result where the student started to feel solely responsible for a potentially hazardous situation, especially because the student noticed that supervisors had recognised potential risks but did not intervene.

I noticed that my supervisors were not very pleased to the situation: meaning long and difficult transport in grounds without any additional help. (S9).

Based on students' descriptions, they or their supervisors did not always see the other healthcare professionals, patients or patients' next of kin as a part of a team. This was shown as a lack of appreciation concerning patients', patients' next of kin or other healthcare professionals' feelings, observations and findings. Through patient safety events, students understand that teamwork in the EMS missions is more than teamwork with your colleague in the ambulance.

Homecare workers clearly had a better knowledge about the patient, and we should have interviewed them better in the situation. (S7).

### 5.4 Feelings related to patient safety events in EMS

Prehospital nursing students' experiences of patient safety events evoked feelings in them during and/or after the event, and the feelings (positive or negative) were an important part of their experiences of patient safety events. In contradictory situations, students felt relief when they experienced patients getting the right treatment or that it was the right choice to transport patients to the hospital. Students had also experienced fear for their own safety or that of others present. The feeling of fear concerning their or others' safety was mainly a result of missing information or some other unexpected element during the task.

From patient safety point of view, I most think about my own safety. You never know where you end up. (S8).

Students were susceptible to the others' feelings especially if the others' feelings were negative. In some cases, students experienced that other healthcare personnel's negative feelings towards the patient or situation caused a threat to the patient's safety. When students experienced the feelings of fear for a patient's safety, it was usually a consequence of some other's (healthcare personnel or emergency response centre operator) actions.

With some other patient, this same kind of mistake could have caused serious consequences, even leading [to] death of the patient. (S16).

Emergency medical services professionals' reactions, behaviour or situations caused feelings of confusion in the students. On the contrary, the situation could be difficult, especially if the patient was in the same age group as the student. Sometimes students' feelings occurred as feelings of self‐blame after the experienced patient safety event or even in the ‘near miss’ situations where they were involved.

I couldn't prepare for the future – I was feeling too good, and my concentration was poor. After that, I was experiencing shame [at] my own actions. (S3).

Even though patient safety events evoked feelings in the students, there were no descriptions of the discussions held by students on their own initiative concerning their feelings during and after the event. Some of the students, however, had experiences of post‐incident defusing sessions as a part of organisational processes.

## 6 DISCUSSION

Our aim was to describe prehospital nursing students' experiences of patient safety culture in emergency medical services during their internship. Descriptions of students' experiences of patient safety events during their internships included positive and negative experiences, but it was more likely that patient safety events were related to negative patient safety incidents. Still, negative experiences could end as positive—for example, in situations where students started to reflect on how they would act in the future when they are fully qualified professionals. This same phenomenon has been reported also in a previous study (Tella et al., 2016).

Students' experiences showed that patient safety culture is clearly a complex phenomenon. As stated before, patient safety culture includes more than just EMS organisations' policies and EMS professionals' technical skills (Mannion & Davies, 2018; Reiman et al., 2010; Sammer et al., 2010). Rather than trying to change the whole patient safety culture at once, students' responses to the question ‘What was the event related to?’ could help EMS and educational organisations to pay attention and develop smaller areas of patient safety culture.

In this study, only one student wrote that a patient safety incident was reported via the healthcare reporting system and none of the students saw that the incident was reported in the patient files. Previously, it has been reported that many EMS personnel had witnessed a patient safety incident, but fewer had reported it (Fisher et al., 2015). The same phenomenon is recognised in other healthcare settings (Tella et al., 2015, 2016). There are also reported barriers to reporting patient safety events in EMS (Sinclair et al., 2018). All of these could indicate that patient safety culture is not so strong that it supports reporting of patient safety events.

### 6.1 Environmental and other unexpected factors in EMS

Experiences of changing working environment and unexpectedness helped the students place patient safety events they experienced in the EMS context. Rather than being a part of a patient safety culture, this contextual framework describes conditions which could affect the shaping of patient safety culture in EMS. Working outside in different conditions, in patients' homes and inside the moving ambulance was part of the external framework in the EMS personnel's working environment. Clearly, the prehospital nursing students recognise that the working environment in EMS is different than in other healthcare settings. However, it was interesting that none of the students describe any events caused by driving even though driving the ambulance has a major role in prehospital care and driving has impacts on safety (Becker & Hugelius, 2021) but also patients' experience of safety (Venesoja et al., 2020).

### 6.2 Working practices and professionalism in EMS

As a part of patient safety culture in EMS, the students experienced poor attitudes or bad behaviour towards patients, relatives, other healthcare professionals and in some cases, towards themselves. Other studies have recognised this same phenomenon (Abelsson & Lindwall, 2017; Dúason, Gunnarsson, et al., 2021; Fisher & Kiernan, 2019; Hörberg et al., 2019; Willassen et al., 2015). However, not all students' experiences concerning behaviour or attitudes were negative. This has been shown also in other studies (Abelsson & Lindwall, 2017; Blomberg et al., 2015). Furthermore, our study shows that students want to absorb good practices and patterns of behaviour. Students recognise bad practices and behaviour, but clearly, they do not want to adopt those models as a part of their own future professionalism. This same phenomenon has also been recognised among new graduate RNs (Hunter & Cook, 2018). Even though working practices and professionalism can be seen as part of a visible manifestation of patient safety culture, this presented theme also shows hidden levels of patient safety culture through attitudes and behaviour behind working practices and ways to use professionalism. Because these hidden levels of patient safety culture can affect patient safety, in future research, it will be crucial to pay attention to these hidden levels and get answers to the question ‘why?’

### 6.3 Teamwork in EMS

In our study, students expressed feeling that they had been a part of a team. However, their experience of being part of the team was not so strong that they had courage to speak up in a situation when they recognised patient safety threats. Similar findings have been showed among medical and nursing students (Alquwez et al., 2019; Bowman et al., 2013; Fisher & Kiernan, 2019). Lack of courage to intervene in a situation may be due to the fear of retribution or punishment or based on team culture itself (Bowman et al., 2013; Fisher & Kiernan, 2019). When we presented our preliminary results to the other prehospital nursing students, they started to reflect on the results to their own experiences of internship in the ambulance and especially teamwork. Teamwork as a phenomenon was discussed the most. They shared their experiences of teamwork in the ambulance and raised a concern about ‘how their instructor can teach and show an example of patient safety teamwork, if they themselves cannot work as a team’. Lack of teamwork, experiences of unsupportive behaviour and loneliness in decision making also emerged when nurses were new in the ambulance service (Hörberg et al., 2017; Hörberg et al., 2019). Descriptions along this theme highlight the importance of the meaning of psychological safety within the team and its effect on patient safety and patient safety culture.

### 6.4 Feelings related to patient safety events in EMS

Patient safety events experienced by prehospital nursing students evoked feelings for them. But it was clear that as a learning environment, EMS does not support tools to handle those feelings, even though feelings are one part of learning (Kolb & Kolb, 2005). A barrier for handling the feelings could be that the students have not experienced the EMS work environment as psychologically safe. On the contrary, students may not have spent enough time in their internships to get familiar with their supervisors or with EMS as a working environment for experiencing psychological safety (Lyman et al., 2020). After all, it is good to notice that feelings not handled properly could cause a risk of second victim phenomenon (Wu et al., 2020). When healthcare workers experience second victim phenomenon, they have a need to talk about the event. Managerial support, but also peer support, plays an important role when healthcare workers cope with an event (Ullström et al., 2014; Zhang et al., 2019). If there is a lack of support, it is more difficult to cope. Worst case, the impact of the event could have long‐lasting consequences (Ullström et al., 2014). Future research is needed to recognise what kind of support (emotional or practical) prehospital nursing students need and whether their needs are similar to EMS‐qualified professionals. We also need future research on how common this second victim phenomenon is among prehospital nursing students.

### 6.5 Limitations

It could be seen as a strength or a limitation that three of the four authors had a history of working in the EMS. Deep experience of working in the EMS and, through that, experiences of prevailing patient safety culture in the EMS help us to understand the students' experiences of patient safety culture in that service. But at the same time, it could cause a lack of openness to the subject. However, one of the researchers had no experience of working in the EMS but had a deep knowledge of patient safety, and this reduced the risk of bias caused by preconceptions of prevailing patient safety culture in EMS. Since researchers had no direct contact list/email addresses of all potential participants (limited in the research permits), we cannot be sure that we have reached all the potential participants. On the contrary, it is possible that invitations have reached students who were not in our target group. These and the data collection method could have caused a risk of self‐selection bias, which means in this study that participants could be those who are interested in or otherwise concerned with this topic.

Because of the data collection method, we have not been able to ask refining questions of the students, which could be seen as a limitation of this study. Regardless of this possible limitation, the data consisted of rich and informative written narratives. Transferability of the results could be limited because we cannot offer information about organisation structures where the students conducted their internships. However, similar phenomena have been recognised in other healthcare settings as well as other EMS settings. Therefore, it is possible that similar results could be found if this study is replicated in other healthcare settings or in other EMS settings.

## 7 CONCLUSIONS

Out of the prehospital nursing students' experiences, and as previous studies have shown, the patient safety culture in EMS is not optimal, and there is a need to develop it. This study shows that prehospital nursing students can recognise patient safety events and the reasons that contributed to those events. Therefore, EMS organisations and managers should use students' observations to develop patient safety culture in EMS.

## 8 RELEVANCE TO CLINICAL PRACTICE

Students are the future employees, so, it is important that managers get information from them as to what cultural manifestations managers should support and what they should try to prevent. Better understanding of how prehospital nursing students have experienced patient safety culture during their internships on ambulances can support educational institutions together with EMS organisations and managers to enhance policies for students expressing their concerns as well as successes of patient safety events. Overall, to develop patient safety, there is a need to enhance speaking‐up culture in the EMS and, through that, to create a psychologically safe environment for both prehospital nursing students and EMS professionals—as well as the patients.

Emergency medical services managers should pay attention to the fact that the patient is not always the only victim in patient safety events. It is possible that if students suffer second victim phenomenon during their internships, it could affect their future careers. To prevent second victim phenomenon and to enhance patient safety, it is important that EMS organisations offer students possibilities to talk over their feelings and concerns. Therefore, it is important that managers in the EMS field lead by example by fostering a teamwork and open speaking culture in their organisations. That is, managers should create psychologically safe, blame‐free and supportive environments where everyone has the courage to speak up, even prehospital nursing students, but also EMS personnel and patients. It is crucial that teachers and course leaders in educational organisations work at teaching psychologically safe teamwork skills and observe how those evolve during learning.

## 10 CONFLICT OF INTEREST

All authors declare that they do not have any competing interests.

## 11 ETHICS STATEMENT

After consultation, Helsinki University Hospital ethics committee stated that this kind of study design do not need a formal ethics approval. However, before the study, we obtained research permits from all three UASs participated in this study (LAB University of Applied Sciences 2020/0002; South Eastern Finland University of Applied Sciences; date14.2.2020; Metropolia University of Applied Sciences id: 24012). Participation was voluntary, and writing and sharing the experience were considered as consent to participate in the study.

12

## Supporting information


Appendix S1

Appendix S2

## Data Availability

The data that support the findings of this study are available from the corresponding author upon reasonable request.

## References

[jocn16396-bib-0001] Abelsson, A. , & Lindwall, L. (2017). What is dignity in prehospital emergency care? Nursing Ethics, 24(3), 268–278. 10.1177/0969733015595544 26260441

[jocn16396-bib-0002] Alquwez, N. , Cruz, J. P. , Alshammari, F. , Felemban, E. M. , Almazan, J. U. , Tumala, R. B. , Alabdulaziz, H. M. , Alsolami, F. , Silang, J. P. B. T. , & Tork, H. M. M. (2019). A multi‐university assessment of patient safety competence during clinical training among baccalaureate nursing students: A cross‐sectional study. Journal of Clinical Nursing, 28(9–10), 1771–1781. 10.1111/jocn.14790 30667103

[jocn16396-bib-0003] Atack, L. , & Maher, J. (2009). Emergency medical and health providers' perceptions of key issues in prehospital patient safety. Prehospital Emergency Care, 14(1), 95–102. 10.3109/10903120903349887 19947873

[jocn16396-bib-0004] Becker, J. , & Hugelius, K. (2021). Driving the ambulance: An essential component of emergency medical services: An integrative review. BMC Emergency Medicine, 21(1), 160–160. 10.1186/s12873-021-00554-9 34922453 PMC8684175

[jocn16396-bib-0005] Bigham, B. L. , Buick, J. E. , Brooks, S. C. , Morrison, M. , Shojania, K. G. , & Morrison, L. J. (2012). Patient safety in emergency medical services: A systematic review of the literature. Prehospital Emergency Care, 16(1), 20–35. 22128905 10.3109/10903127.2011.621045

[jocn16396-bib-0006] Blomberg, A.‐C. , Willasen, E. , von Post, I. , & Lindwall, L. (2015). Student nurses' experiences of preserved dignity in perioperative practice: Part 1, 22, 676–687. 10.1177/0969733014542675 25106458

[jocn16396-bib-0007] Bowman, C. , Neeman, N. , & Sehgal, N. L. (2013). Enculturation of unsafe attitudes and behaviors: Student perceptions of safety culture. Academic Medicine, 88(6), 802–810. 10.1097/ACM.0b013e31828fd4f4 23619067 PMC4024094

[jocn16396-bib-0008] Braun, V. , & Clarke, V. (2006). Using thematic analysis in psychology. Qualitative Research in Psychology, 3(2), 77–101.

[jocn16396-bib-0009] Brooks, J. , Grugulis, I. , & Cook, H. (2020). Rethinking situated learning: Participation and communities of practice in the UKfire and rescue service. Work, Employment and Society, 34(6), 1045–1061.

[jocn16396-bib-0010] Dúason, S. , Ericsson, C. , Jónsdóttir, H. L. , Andersen, J. V. , & Andersen, T. L. (2021). European paramedic curriculum—A call for unity in paramedic education on a European level. Scandinavian Journal of Trauma, Resuscitation and Emergency Medicine, 29(1), 1–4. 34059084 10.1186/s13049-021-00889-z PMC8166099

[jocn16396-bib-0011] Dúason, S. , Gunnarsson, B. , & Svavarsdóttir, M. H. (2021). Patient handover between ambulance crew and healthcare professionals in Icelandic emergency departments: A qualitative study. Scandinavian Journal of Trauma, Resuscitation and Emergency Medicine, 29(1), 21–21. 10.1186/s13049-021-00829-x 33509266 PMC7842055

[jocn16396-bib-0012] Edmonson, A. (2004). Psychological safety, trust, and learning: A group‐level lens. In Trust and distrust in organizations: Dilemmas and approaches, edited by Kramer R. & Cook K. pp. 239‐272. New York: Russell Sage Foundation.

[jocn16396-bib-0013] Fisher, J. D. , Freeman, K. , Clarke, A. , Spurgeon, P. , Smyth, M. , Perkins, G. D. , Sujan, M.‐A. , & Cooke, M. W. (2015). Patient safety in ambulance services: A scoping review. Health Services and Delivery Research, 3(21), 1–250. 10.3310/hsdr03210 25996021

[jocn16396-bib-0014] Fisher, M. , & Kiernan, M. (2019). Student nurses' lived experience of patient safety and raising concerns. Nurse Education Today, 77, 1–5. 10.1016/j.nedt.2019.02.015 30877869

[jocn16396-bib-0015] Flanagan, J. C. (1954). The critical incident technique. Psychological Bulletin, 51(4), 327–358. 13177800 10.1037/h0061470

[jocn16396-bib-0016] Forsell, L. , Forsberg, A. , Kisch, A. , & Rantala, A. (2020). Specialist ambulance Nurses' perceptions of nursing: A Phenomenographic study. International Journal of Environmental Research and Public Health, 17(14), 5018. 10.3390/ijerph17145018 32668619 PMC7400022

[jocn16396-bib-0017] Hagiwara, M. A. , Magnusson, C. , Herlitz, J. , Seffel, E. , Axelsson, C. , Munters, M. , Strömsöe, A. , & Nilsson, L. (2019). Adverse events in prehospital emergency care: A trigger tool study. BMC Emergency Medicine, 19(1), 14. 10.1186/s12873-019-0228-3 30678636 PMC6345067

[jocn16396-bib-0018] Holmberg, M. , Fagerberg, I. , & Wahlberg, A. C. (2017). The knowledge desired by emergency medical service managers of their ambulance clinicians – A modified Delphi study. International Emergency Nursing, 34, 23–28. 10.1016/j.ienj.2017.03.007 28545930

[jocn16396-bib-0019] Hunter, K. , & Cook, C. (2018). Role‐modelling and the hidden curriculum: New graduate nurses' professional socialisation. Journal of Clinical Nursing, 27(15–16), 3157–3170. 10.1111/jocn.14510 29752850

[jocn16396-bib-0020] Hörberg, A. , Lindström, V. , Kalén, S. , Scheja, M. , & Vicente, V. (2017). Striving for balance – A qualitative study to explore the experiences of nurses new to the ambulance service in Sweden. Nurse Education in Practice, 27, 63–70. 10.1016/j.nepr.2017.08.015 28846965

[jocn16396-bib-0021] Hörberg, A. , Lindström, V. , Scheja, M. , Conte, H. , & Kalén, S. (2019). Challenging encounters as experienced by registered nurses new to the emergency medical service: Explored by using the theory of communities of practice. Advances in Health Sciences Education: Theory and Practice, 24(2), 233–249. 10.1007/s10459-018-9862-x 30443693 PMC6483944

[jocn16396-bib-0022] Kolb, A. Y. , & Kolb, D. A. (2005). Learning styles and learning spaces: Enhancing experiential learning in higher education. Academy of Management Learning & Education, 4(2), 193–212.

[jocn16396-bib-0023] LAB . (2022). Curricula: Bachelor's degree Programme in emergency care. LAB University of Applied Sciences.

[jocn16396-bib-0024] Labrague, L. J. (2021). Influence of nurse managers' toxic leadership behaviours on nurse‐reported adverse events and quality of care. Journal of Nursing Management, 29(4), 855–863. 10.1111/jonm.13228 33617119

[jocn16396-bib-0025] Lederman, J. , Lindström, V. , Elmqvist, C. , Löfvenmark, C. , Ljunggren, G. , & Djärv, T. (2021). Non‐conveyance of older adult patients and association with subsequent clinical and adverse events after initial assessment by ambulance clinicians: A cohort analysis. BMC Emergency Medicine, 21(1), 1–11. 34895152 10.1186/s12873-021-00548-7 PMC8666056

[jocn16396-bib-0026] Lyman, B. , Gunn, M. M. , & Mendon, C. R. (2020). New graduate registered nurses' experiences with psychological safety. Journal of Nursing Management, 28(4), 831–839. 10.1111/jonm.13006 32173958

[jocn16396-bib-0027] Mannion, R. , & Davies, H. (2018). Understanding organisational culture for healthcare quality improvement. BMJ, 363, k4907. 10.1136/bmj.k4907 30487286 PMC6260242

[jocn16396-bib-0028] Metropolia . (2022). Curricula: Degree program in emergency care. Metropolia University of Applied Sciences.

[jocn16396-bib-0029] Paramedics, T. C.O. (2019). Paramedic curriculum guidance (5th ed.). The College of Paramedics.

[jocn16396-bib-0030] Paulin, J. , Kurola, J. , Koivisto, M. , & Iirola, T. (2021). EMS non‐conveyance: A safe practice to decrease ED crowding or a threat to patient safety? BMC Emergency Medicine, 21(1), 115. 10.1186/s12873-021-00508-1 34627138 PMC8502399

[jocn16396-bib-0031] Péculo‐Carrasco, J. A. , De Sola, H. , Casal‐Sánchez, M. d. M. , Rodríguez‐Bouza, M. , Sánchez‐Almagro, C. P. , & Failde, I. (2020). Feeling safe or unsafe in prehospital emergency care: A qualitative study of the experiences of patients, carers and healthcare professionals. Journal of Clinical Nursing, 29(23–24), 4720–4732. 10.1111/jocn.15513 32979872

[jocn16396-bib-0032] Reiman, T. , Pietikainen, E. , & Oedewald, P. (2010). Multilayered approach to patient safety culture. Quality & Safety in Health Care, 19(5), e20. 10.1136/qshc.2008.029793 20724396

[jocn16396-bib-0033] Sammer, C. E. , Lykens, K. , Singh, K. P. , Mains, D. A. , & Lackan, N. A. (2010). What is patient safety culture? A review of the literature: Patient safety culture. Journal of Nursing Scholarship, 42(2), 156–165. 10.1111/j.1547-5069.2009.01330.x 20618600

[jocn16396-bib-0034] Sinclair, J. E. , Austin, M. A. , Bourque, C. , Kortko, J. , Maloney, J. , Dionne, R. , … Calder, L. A. (2018). Barriers to self‐reporting patient safety incidents by paramedics: A mixed methods study. Prehospital Emergency Care, 22(6), 762–772. 10.1080/10903127.2018.1469703 29787325

[jocn16396-bib-0035] Sjölin, H. , Lindström, V. , Hult, H. , Ringsted, C. , & Kurland, L. (2019). Common core content in education for nurses in ambulance care in Sweden, Finland and Belgium. Nurse Education in Practice, 38, 34–39. 10.1016/j.nepr.2019.05.017 31176241

[jocn16396-bib-0036] Steven, A. , Pearson, P. , Turunen, H. , Myhre, K. , Sasso, L. , Vizcaya‐Moreno, M. F. , Pérez‐Cañaveras, R. M. , Sara‐Aho, A. , Bagnasco, A. , Aleo, G. , Patterson, L. , Larkin, V. , Zanini, M. , Porras, J. , Khakurel, J. , Azimirad, M. , Ringstad, Ø. , Johnsen, L. , Haatainen, K. , … Tella, S. (2021). Development of an international tool for students to record and reflect on patient safety learning experiences. Nurse Educator, 47, E62–E67. 10.1097/NNE.0000000000001142 34882101

[jocn16396-bib-0037] Steven, A. , Tella, S. , Turunen, H. , Flores Vizcaya‐Moreno, M. , Pérez‐Cañaveras, R. M. , Porras, J. , Bagnasco, A. , Sasso, L. , Myhre, K. , Sara‐Aho, A. , Ringstad, Ø. , & Pearson, P. (2019). Shared learning from national to international contexts: A research and innovation collaboration to enhance education for patient safety. Journal of Research in Nursing, 24(3–4), 149–164. 34394520 10.1177/1744987118824628 PMC7932281

[jocn16396-bib-0038] Steven, A. , Wilson, G. , Turunen, H. , Vizcaya‐Moreno, M. F. , Azimirad, M. , Kakurel, J. , Porras, J. , Tella, S. , Pérez‐Cañaveras, R. , Sasso, L. , Aleo, G. , Myhre, K. , Ringstad, Ø. , Sara‐Aho, A. , Scott, M. , & Pearson, P. (2020). Critical incident techniques and reflection in nursing and health professions education: Systematic narrative review. Nurse Educator, 45(6), E57–E61. 10.1097/NNE.0000000000000796 31972840

[jocn16396-bib-0039] Suri, H. (2011). Purposeful sampling in qualitative research synthesis. Qualitative Research Journal, 11, 63–75.

[jocn16396-bib-0040] Tella, S. , Smith, N.‐J. , Partanen, P. , & Turunen, H. (2016). Work placements as learning environments for patient safety: Finnish and British preregistration nursing students' important learning events. Journal of Vocational Education & Training, 68(1), 51–69. 10.1080/13636820.2015.1104715

[jocn16396-bib-0041] Tella, S. , Smith, N. J. , Partanen, P. , Jamookeeah, D. , Lamidi, M. L. , & Turunen, H. (2015). Learning to ensure patient safety in clinical settings: Comparing Finnish and British nursing students' perceptions. Journal of Clinical Nursing, 24(19–20), 2954–2964. 26216053 10.1111/jocn.12914

[jocn16396-bib-0042] Tong, A. , Sainsbury, P. , & Craig, J. (2007). Consolidated criteria for reporting qualitative research (COREQ): A 32‐item checklist for interviews and focus groups. International Journal for Quality in Health Care, 19(6), 349–357. 10.1093/intqhc/mzm042 17872937

[jocn16396-bib-0043] Ullström, S. , Andreen Sachs, M. , Hansson, J. , Øvretveit, J. , & Brommels, M. (2014). Suffering in silence: A qualitative study of second victims of adverse events. BMJ Quality & Safety, 23(4), 325–331. 10.1136/bmjqs-2013-002035 PMC3963543 24239992

[jocn16396-bib-0044] Venesoja, A. , Castrén, M. , Tella, S. , & Lindström, V. (2020). Patients' perceptions of safety in emergency medical services: An interview study. BMJ Open, 10(10), e037488. 10.1136/bmjopen-2020-037488 PMC7580042 33087370

[jocn16396-bib-0045] Wankhade, P. , & Brinkman, J. (2014). The negative consequences of culture change management: Evidence from a UKNHS ambulance service. The International Journal of Public Sector Management, 27(1), 2–25. 10.1108/IJPSM-05-2012-0058

[jocn16396-bib-0046] Willassen, E. , Blomberg, A.‐C. , von Post, I. , & Lindwall, L. (2015). Student nurses' experiences of undignified caring in perioperative practice – Part II. Nursing Ethics, 22(6), 688–699. 10.1177/0969733014542678 25106457

[jocn16396-bib-0047] World Medical Association . (2013). World medical association declaration of Helsinki: Ethical principles for medical research involving human subjects. JAMA, 310(20), 2191–2194. 24141714 10.1001/jama.2013.281053

[jocn16396-bib-0048] Wu, A. W. , Shapiro, J. , Harrison, R. , Scott, S. D. , Connors, C. , Kenney, L. , & Vanhaecht, K. (2020). The impact of adverse events on clinicians: Whatʼs in a name? Journal of Patient Safety, 16(1), 65–72. 10.1097/PTS.0000000000000256 29112025

[jocn16396-bib-0049] Yardley, S. , Teunissen, P. W. , & Dornan, T. (2012). Experiential learning: Transforming theory into practice. Medical Teacher, 34(2), 161–164. 22288996 10.3109/0142159X.2012.643264

[jocn16396-bib-0050] Zhang, X. , Li, Q. , Guo, Y. , & Lee, S. Y. (2019). From organisational support to second victim‐related distress: Role of patient safety culture. Journal of Nursing Management, 27(8), 1818–1825. 10.1111/jonm.12881 31556205

