# Supplementary material for: Prehospital nursing students' experiences of patient safety culture in emergency medical services—A qualitative study
Source: J Clin Nurs. 2022 Jun 7;32(5-6):847–58. doi: 10.1111/jocn.16396 (PMC10083998; doi:10.1111/jocn.16396)
Supplement: Supplementary file 2 — Appendix S2 [file JOCN-32-847-s002.docx]

Supplementary file 2: Example of the coding tree.

| **Students’ description** | **Code** | **Collated extracts** | **Theme** |
| --- | --- | --- | --- |
| *‘We turn the man to his back and then we saw a gun in his hand. Quickly we string together that the patient has [been] shot in his head’* | Unexpected situation caused by patient previous action | Expect the unexpected | **Environmental and other unexpected factors in EMS** |
| *‘I drew attention to the importance of reporting and how to confirm that recipient has [understood] that. Sometimes it goes that even [if] you give comprehensive report, it is not listened or internalised‘.* | Working practices: Asking and receiving information | Communication and information flow | **Working practices and professionalism in EMS** |
| *After the event, we first discuss it together with my supervisor, who told me that I was acting right when I followed the woman. It was nice to get positive feedback for my own performance’.* | Experiences of teamwork: Situation was discussed as a team | Discussions after the EMS task with the supervisors/others present | **Teamwork in EMS** |
| *‘With some other patient, this same kind of mistake could have caused serious consequences, even leading [to] death of the patient‘.* | Feelings: Fear of patient's safety | Second victim phenomenon associated with other caregivers’ actions | **Feelings related to patient safety events in EMS** |
